# The effect of material defect orientation on rolling contact fatigue of a ball bearing

**DOI:** 10.1038/s41598-023-42677-y

**Published:** 2023-09-16

**Authors:** Akbar Ghazanfari Holagh, Javad Alizadeh Kaklar

**Affiliations:** Department of Mechanical Engineering, Urmia University, Urmia, Iran

**Keywords:** Mechanical engineering, Metals and alloys

## Abstract

In this study, finite element method (FEM) is applied to investigate the effect of material defect orientation on subsurface crack initiation in rolling bearings. First, the history of stress intensity factors (SIFs) of a subsurface cubic material defect is calculated. SIFs calculation is conducted when it is parallel to the rolling surface of a bearing ring ($\varphi = 0^\circ$) under a ball rolling on the ring. Then, the effect of defect orientation, varying from $\varphi = 0^\circ$ to $\varphi \; = \;30,\; 45\; {\text{and}} \;60^\circ$, on the history of SIFs is investigated. It is deduced that changing the defect orientation results in a threefold increase in the equivalent SIF range compared to parallel orientation.

## Introduction

**Figure 1:**
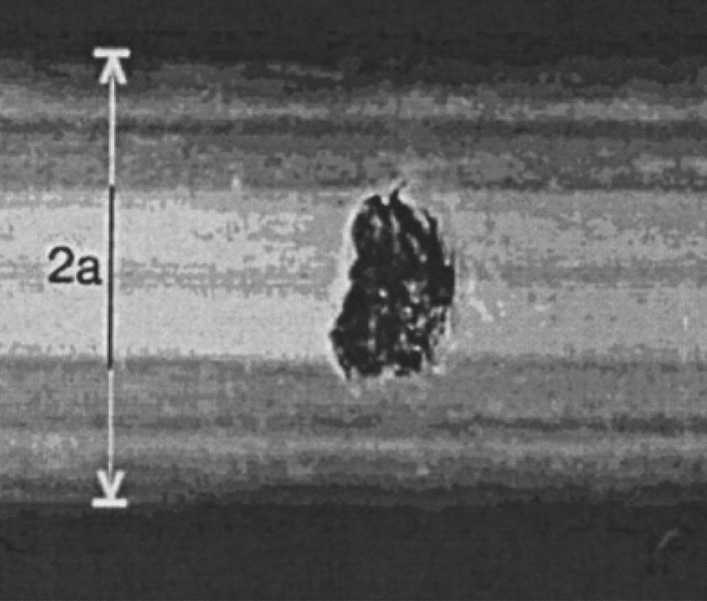
Spalling and surface roughness of a bearing due to the RCF^1^.

Bearings are mechanical elements that reduce friction and constrain the relative motion of machine components. The prediction of bearing failure is a critical concern in the industry due to the potential for unexpected failures, which can result in significant costs. Rolling contact fatigue (RCF) is recognized as one of the most significant failure modes affecting rolling bearings. Within these bearings, the occurrence of rolling contact fatigue (RCF) can contribute to the nucleation and propagation of subsurface cracks, ultimately resulting in spalling, shelling, surface roughness, and eventual bearing failure (see Fig. 1). The initiation of subsurface cracks under RCF conditions is mainly due to the incidence of maximum shearing stresses and the existence of material defects below the contact surface.

In a ball bearing, the rolling motion of the balls against the inner and outer rings subjects a very small volume of the material to repetitive stresses. Under such alternating stresses, the presence of subsurface material defects can nucleate subsurface cracks. In general, the existence of a material defect in the inner ring, outer ring, or balls accelerates the failure of the bearing, causing substantial economic costs. Indeed, material defects within the bearing result in a significant concentration of stress around them, rendering the bearing more susceptible to the initiation of damage. Several studies have investigated the effect of material defects on crack initiation and propagation under RCF conditions^2–6^.

Among the first studies on crack initiation under RCF conditions, Way^7^ observed that these cracks grow in the first stage under the shear mode and directly at an angle range of 10°–30° with respect to the rolling surface. Then, in the second stage, the cracks were found to further grow in a curved shape under the opening mode. Keer et al.^8^ presented numerical results for a horizontal subsurface crack in an elastic half-space under rolling contact loading.

Melander^9^ employed the Finite Element Method (FEM) to determine the crack driving force, considering crack tip displacement and energy release rate, for short cracks located at inclusions within bearing steel under the influence of rolling contact loading. It was found that the tendency to initiate cracks at the location of material defects might have a significant influence on the fatigue life. Hashimoto et al.^10^ examined the effects of material defects on internal fracture-type rolling contact fatigue life. They introduced hot isostatic pressing as a highly useful technique for closing the pores and thereby, improving the RCF life of bearing steels. Also, in another study, a series of experiments were carried out by Hashimoto et al.^11^ to evaluate the impact of sulfide defects on crack initiation and RCF life of bearing steels. Murakami^12^ looked into the influence of material defects on fatigue design. The study suggested a new efficient and reliable inclusion rating method for high-strength steels based on the statistics of extremes using hydrogen embrittlement phenomenon.

Among the more recent studies on the defect effects on RCF strength is the one conducted by Deng et al.^13^. The authors assessed the influence of the position of elliptical-shaped subsurface cracks on the fatigue crack growth in a bearing ring under RCF conditions. In another study, Deng et al.^14^ used microscopic observations to evaluate the effect of inclusions and inclusion hardness on crack initiation in bearings. The influence of the size and depth of the small defects on the strength of bearing steel was investigated by Hisao et al.^15^; the authors reported that fatigue cracks initiate at the edge near the bottom of the pore and then, propagate by shear modes. The mechanisms improving fatigue life in remanufactured bearings under RCF conditions by continuum damage mechanics were scrutinized by Paulson et al.^16^. In a similar research, Golmohammadi et al.^17^ employed a coupled continuum damage mechanics as well as finite element model to simulate RCF under high loading conditions. It was concluded that as the cycles increases, contact width grows as well; this was explained by the plastic strain accumulation. The impact of material defects on the fatigue limit of bearing steels under RCF conditions was investigated by Brian et al.^18^. The findings demonstrated that the geometric effect a material defect on the fatigue limit is relatively minimal. Ghajar and Alizadeh Kaklar^19^ and Abdoli et al.^20^ respectively derived weight functions for two-dimensional and one-dimensional subsurface cracks, which may originate from a material defect under RCF conditions. Regarding the availability of the weight function for a crack, its condition can be easily evaluated by calculating stress intensity factors. In this respect, considering the shear fracture modes, Samadlu and Alizadeh Kaklar^21^ studied the fatigue crack growth pattern of a two-dimensional subsurface crack subjected to moving contact pressure. Liu et al.^22^ analyzed the effect of subsurface cracks on the contact characteristics of a roller bearing. The authors, also, extracted the relation between the contact area specifications and the crack sizes. The influence of interacting small defects on the fatigue limit of bearing steel was experimentally evaluated by Aman et al.^23^. The study highlighted significant variations in the non-propagating crack characteristics and the severity of crack coalescence across different materials. Hua et al.^3^ modeled a material defect as a cubic cavity in parallel to the rolling surface of a ball bearing; the authors also investigated the effects of a material defect on crack propagation under RCF conditions. It was concluded that the shear mode is dominant for crack propagation.

As the literature survey unveils, several studies have explored the impact of parameters such as defect size, depth, and geometry on fatigue life. However, the effect of material defect orientation on macro-crack initiation has remained unclear. To fill this gap, the present study aims to investigate the impact of an internal material defect orientation on the initiation of macro cracks under RCF conditions in the ring of a ball bearing. To this end, the authors conducted a three-dimensional finite element modeling of a cubic material defect at the subsurface of a bearing ring (such as then one explained in Ref.^3^), employing ABAQUS finite element commercial software. The four edges of the cubic defect aligned along the axial direction of the ring are considered as four line-cracks. The validity of the finite element model was established by comparing the maximum contact stress values obtained from the model with those predicted by Hertzian contact theory. The validated FE model is used to determine the history of the mixed mode stress intensity factors for different defect orientations. Note that the material defect orientation is defined as the angle of the top surface of the defect with the contact surface of the bearing ring. The range of Modes I, II, and III stress intensity factors and the Tanaka equivalent range of stress intensity factors are determined using the history of Modes I, II, and III as well as the stress intensity factors of the material defect. Moreover, the effect of the defect orientation on the tendency of crack growth from the location of the edges of the defect and the formation of a macro-crack is evaluated.

## Finite element modeling of a defective ball-bearing

**Figure 2:**
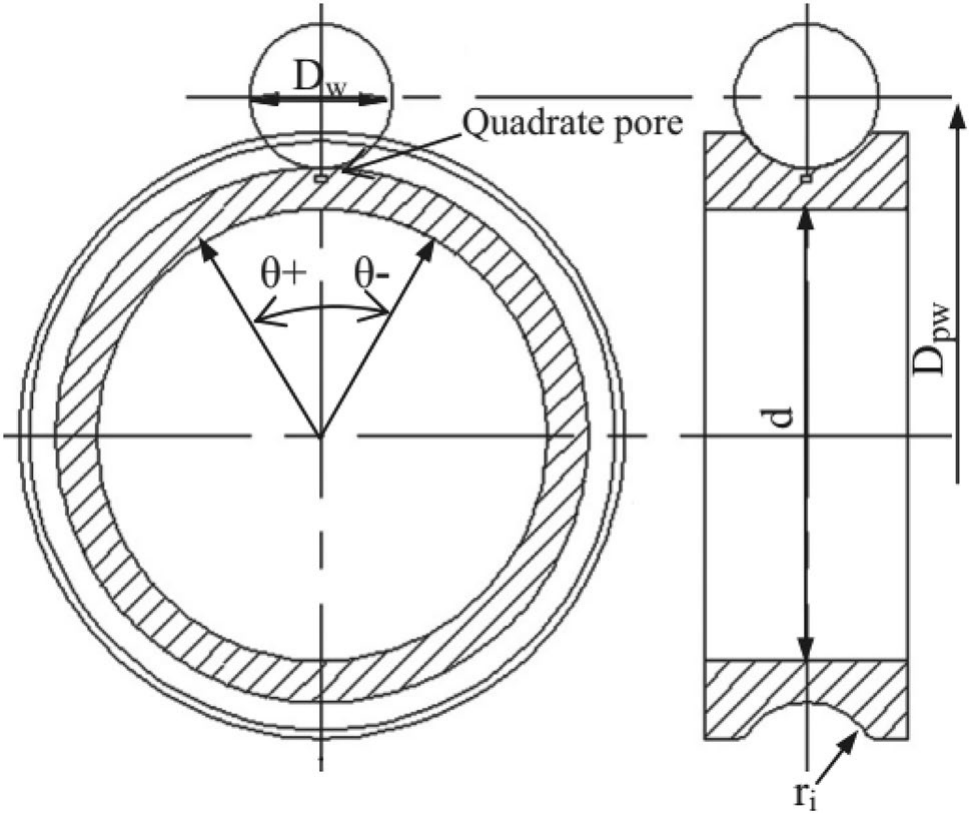
Plan of the ball, inner ring, and material defect of a ball bearing^3^.

In this paper, the inner ring of a 6208 (bearing designation, SKF) deep groove ball bearing is considered as the RCF research object. To reduce the computational time required, the modeling and problem-solving steps were streamlined by focusing solely on the inner ring and one of the balls. The ball is defined as a three-dimensional rigid part, and the ring is a three-dimensional deformable solid. A plan of the ring and ball geometry used in this research is presented in Fig. 2, and its geometric dimensions are represented in Table 1. The symbol $\theta$ indicates the rotation angle of the inner ring. For a fix ball, the rotation angle is defined to be negative when the contact position is to the left of the pore.

**Table 1:** Basic geometrical data of a 6208 deep groove ball bearing^3^.

Name	Symbol	Value (mm)
Bearing bore diameter	d	40
Bearing width	B	18
Steel ball diameter	D_w_	12.7
Distance of the balls center	D_pw_	60
Inner raceway groove curvature radius	r_i_	6.54

**Figure 3:**
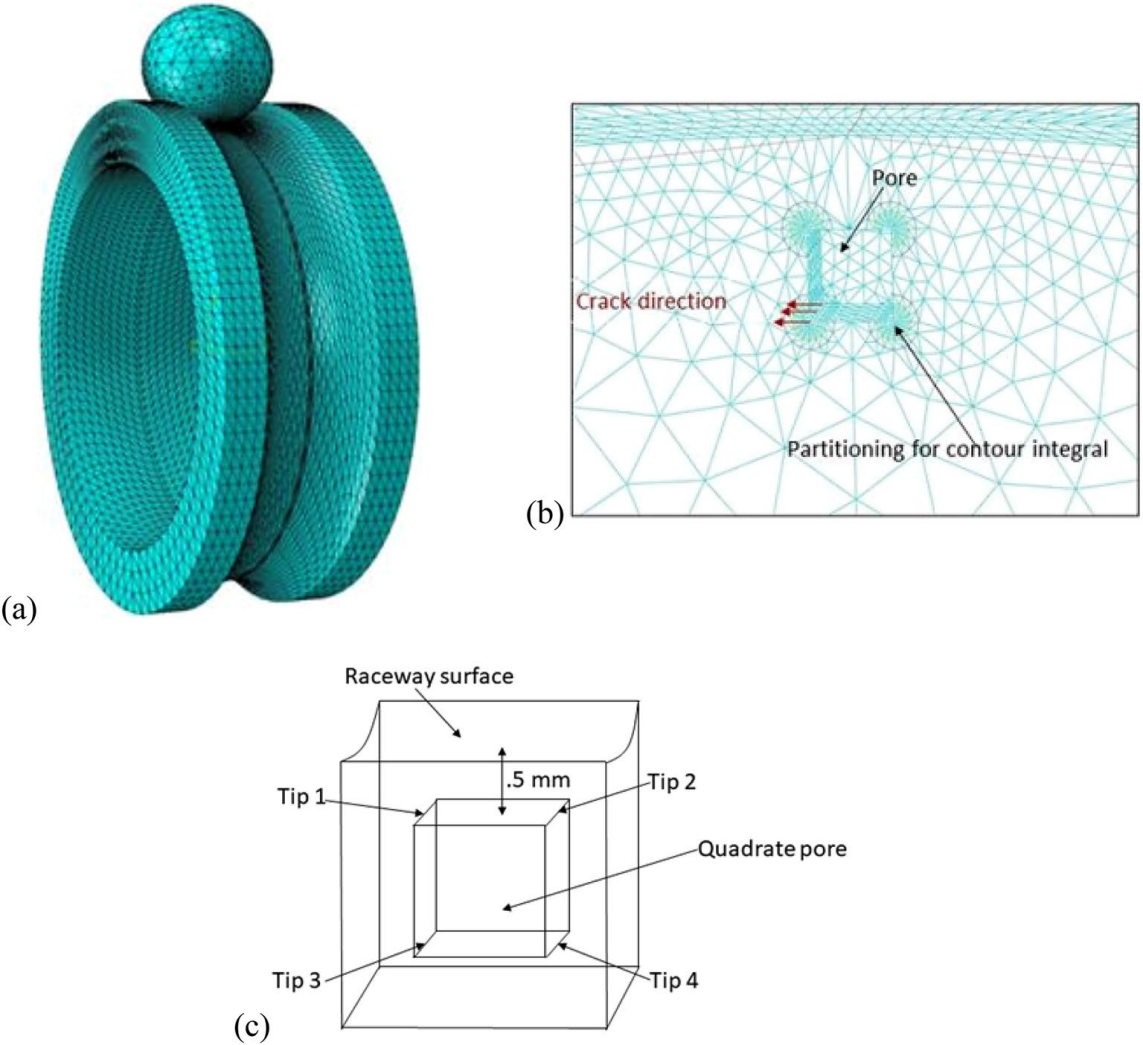
(**a**) Finite element model of the ball/ring contact, (**b**) meshing of the defect around the area, and (**c**) the crack tips of the pore.

To minimize the runtime and achieve more precise outcomes, it is imperative to employ small elements, approximately 0.12 $\mathrm{mm}$ in size, in the contact region between the ball and the ring, as well as around the defect. Furthermore, in areas of the finite element model that are farther away from the contact region and where stress values are close to zero, coarser elements are utilized to further optimize the computational efficiency. The developed FE model is displayed in Fig. 3; the ring and ball are meshed using 78,000 elements of C3D10 type. The material defect is modeled as a cubic pore located under the surface of the inner ring groove and parallel to the rolling surface. The pore dimension is 0.5 mm, and it is located at a depth of 0.5 mm in the middle of the ring groove. The material used in this study is AISI 52100 steel (Young’s modulus = 210 GPa, Poisson’s ratio = 0.3, and material density = 7850 kg/m^3^). To study the effect of the pore on the crack initiation, we defined the crack front, crack tip, and the direction of crack extension (Fig. 3b). To this end, four crack tips are defined, as shown in Fig. 3c. Tips 1 and 2 are defined as top cracks and Tips 3 and 4 are defined as down cracks.

The solving method of the model is chosen statically. That is, the dynamic effects of the ball movement on the ring are neglected. Also, ball/ring interaction is defined as surface-to-surface contact. In this definition, the groove surface of the ring is defined as the master surface according to the smaller element size, and the ball is considered as the slave surface. The coefficient of friction for the ball/ring contact is assumed to be 0.15^3^. Self-contact is defined between the internal surfaces of the pore. By coupling the bearing bore surface and a reference point located at the bearing center, a 1 kN radial force is exerted at the reference point toward the center of the ball. All six degrees of freedom for the ball are restricted. Furthermore, the ring is constrained in all directions except for radial displacement to facilitate the pressing of the ball against the ring.

**Figure 4:**
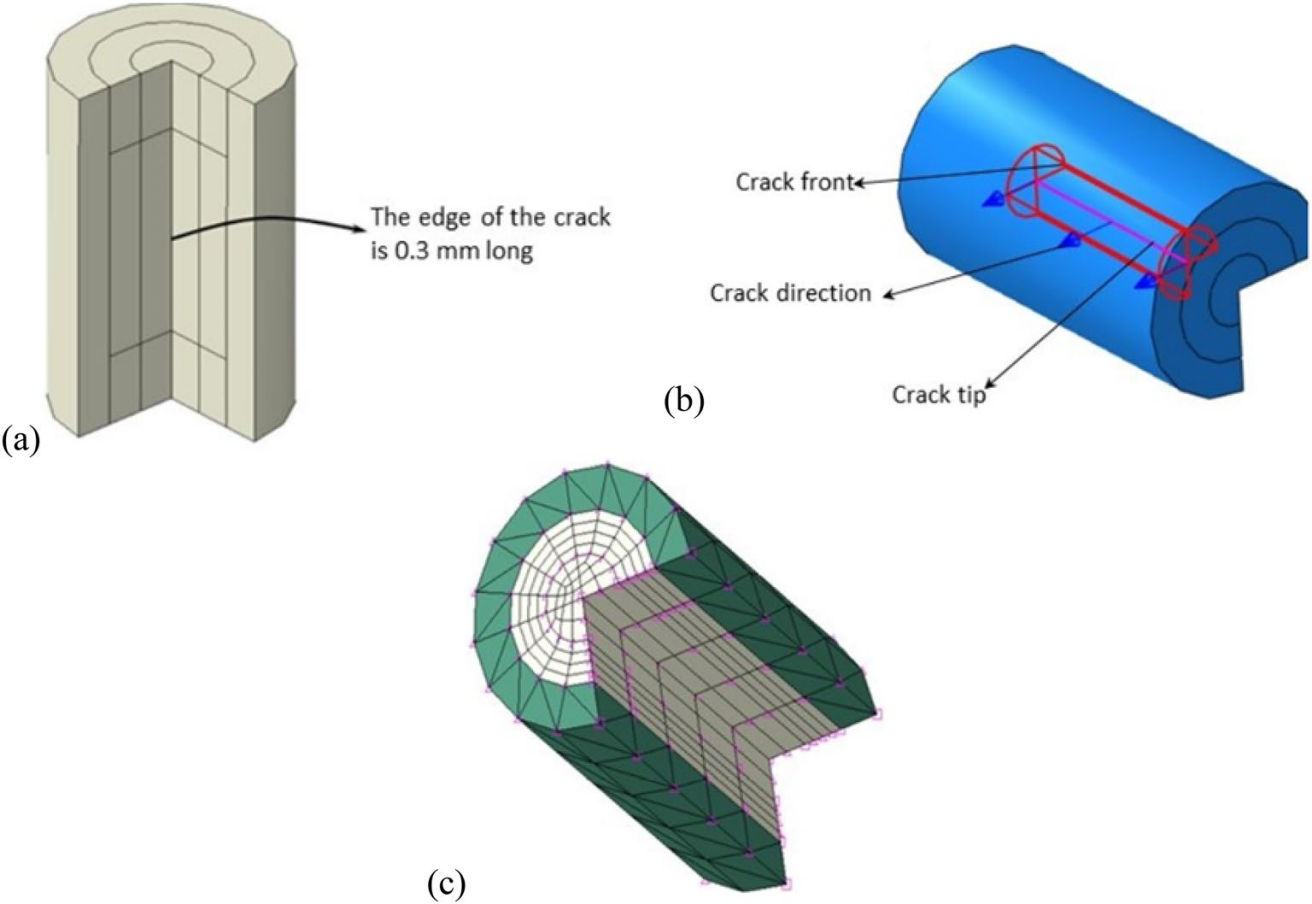
(**a**) Partitions used for the around of cube edge, (**b**) definitions of the crack tip, crack front, and direction of crack extension for crack 1, and (**c**) contours and meshing for the crack around the area.

The contour integration method is used for calculating the stress intensity factors. The most important aspect of finite element modeling is meshing and crack defining for the defect such that the model can be solved without errors. Crack tips and contours are partitioned as shown in Fig. 4. According to Fig. 4a, the middle part of the cube edge with a length of 0.3 mm is defined as the crack tip. Besides, the two sides of the edge are separated by a length of 0.1 mm each; this is done for the compatibility of the elements and the possibility of calculating the contour integrals. The use of two arc partitions with radii of 0.05 mm and 0.1 mm around the crack edge allows for calculating the stress intensity factors on four contours. The first partition is considered as the crack front and the first contour, and the second partition is taken into account as the second to fourth contours, as depicted in Fig. 4b. To complete the crack definition and specify the directions that mode I, II and III SIFs are calculated, crack extension direction (**q** vector) is defined. The meshing of the crack tip around the area is indicated in Fig. 4c. Outside the contours, an arc partition with a radius of 0.15 mm is used to facilitate the uniform growth of the elements’ size.

## Validation of the finite element model

**Figure 5:**
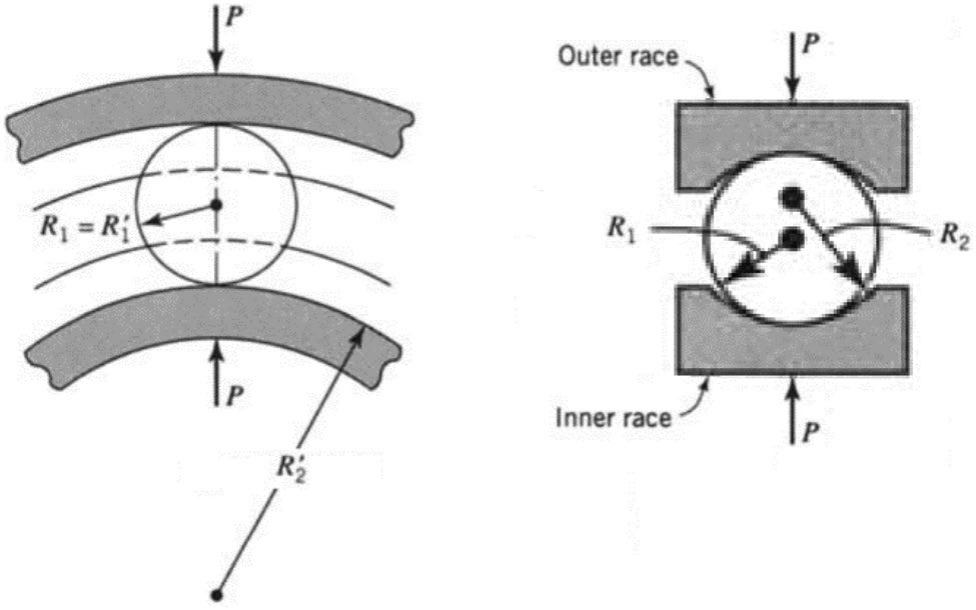
Radii of the principal curvatures for the members in contact^24^.

**Figure 6:**
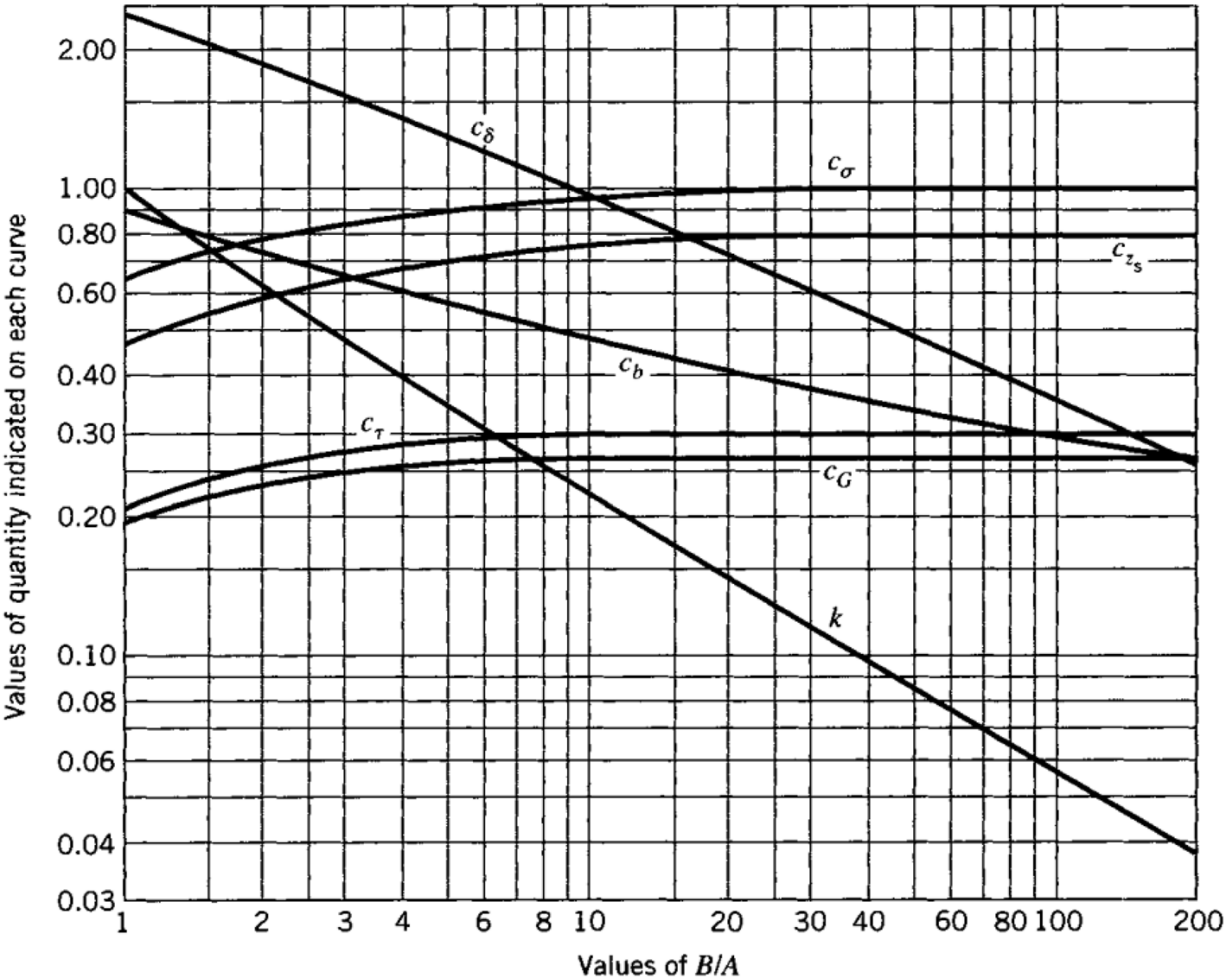
Values of $c_{b}$, $k$ and $c_{\sigma }$ with respect to $B/A$ ratio^24^.

According to Hertzian contact theory^24^, the contact of two rolling members under the compression force, P, results in an elliptical contact area. The dimensions of the contact ellipse and the maximum contact stress occurring in the center of the contact ellipse are given by Eqs. (1)–(5)^24^:

1 $$\begin{gathered} A = \frac{1}{4}\left( {\frac{1}{{R_{1} }} + \frac{1}{{R_{2} }} + \frac{1}{{R^{\prime}_{1} }} + \frac{1}{{R^{\prime}_{2} }}} \right) - \frac{1}{4}\left| {\frac{1}{{R_{1} }} - \frac{1}{{R^{\prime}_{1} }} + \frac{1}{{R_{2} }} - \frac{1}{{R^{\prime}_{2} }}} \right| \hfill \\ B = \frac{1}{4}\left( {\frac{1}{{R_{1} }} + \frac{1}{{R_{2} }} + \frac{1}{{R^{\prime}_{1} }} + \frac{1}{{R^{\prime}_{2} }}} \right) + \frac{1}{4}\left| {\frac{1}{{R_{1} }} - \frac{1}{{R^{\prime}_{1} }} + \frac{1}{{R_{2} }} - \frac{1}{{R^{\prime}_{2} }}} \right| \hfill \\ \end{gathered}$$

2 $$\Delta = \frac{1}{A + B}\left( {\frac{{1 - \nu_{1}^{2} }}{{E_{1} }} + \frac{{1 - \nu_{2}^{2} }}{{E_{2} }}} \right)$$

3 $$b = c_{b} \sqrt[3]{P\Delta }$$

4 $$a = \frac{b}{k}$$

5 $$\sigma_{max} = c_{\sigma } \frac{b}{\Delta }$$

where $R_{1}$ and $R^{\prime}_{1}$ are the radiuses of principal curvatures in the surface of member 1, whereas $R_{2}$ and $R^{\prime}_{2}$ are the radiuses of principal curvatures in the surface of member 2. These radii are presented in Fig. 5 for the ball and ring curvatures. Also, $E_{1}$ and $E_{2}$ signify the elastic modulus, and $\nu_{1}$ and $\nu_{2}$ represent the Poisson’s ratio of the members 1 and 2, respectively. Furthermore, *a*, *b*, and $\sigma_{max}$ denote the major and minor semi-axes of the contact ellipse, and the maximum contact stress, respectively. Finally, $c_{b}$, *k*, and $c_{\sigma }$ represent the constants exhibited in the diagram of Fig. 6.

Substituting geometrical and mechanical properties of the ball and ring in Eqs. (1) and (2) gives:

$${\text{A}} = 2.287\frac{1}{{\text{m}}}$$

$${\text{B}} = 99.881\frac{1}{{\text{m}}}$$

$$\Delta = 4.241 \times 10^{ - 14} \frac{{{\text{m}}^{3} }}{{\text{N}}}$$

Then, the constants of Eqs. (3)–(5) are obtained for $B/A=43.673$ using the diagram of Fig. 6.

$$c_{b} = 0.343$$

$$k = 0.0914$$

$$c_{\sigma } = 1$$

Finally, contact area dimensions and maximum contact stress are calculated for *P* = 1 kN by Eqs. (3)–(5) as follow:

$$b = 0.119 \;{\text{mm}}$$

$$a = 1.308\;{\text{ mm}}$$

$$\sigma_{max} = 2890\; {\text{MPa}}$$

**Figure 7:**
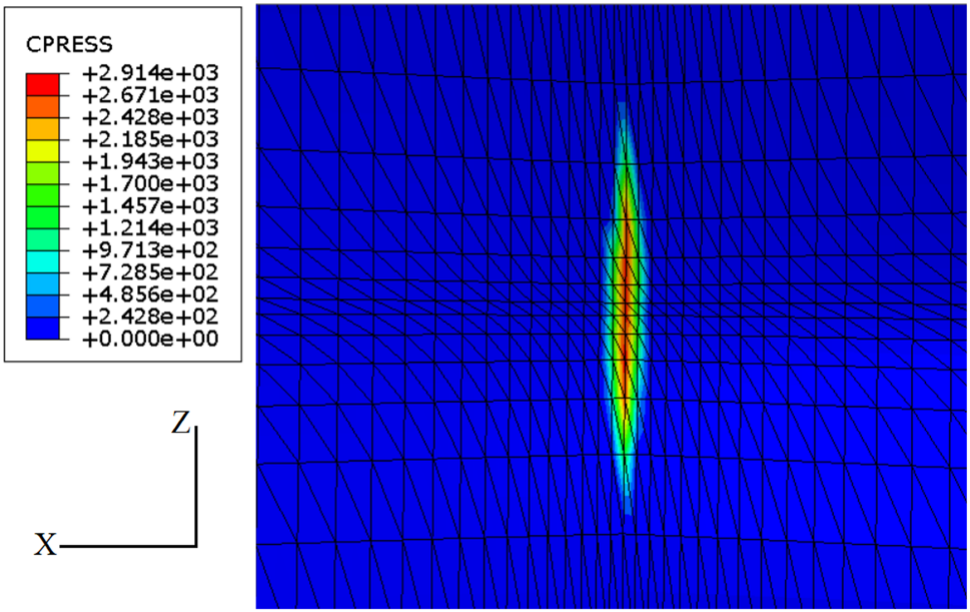
Contact stress distribution of the bearing ring obtained by the FE solution. X and Z axes are in circumferential and axial directions, respectively.

Figure 7 shows the finite element results for contact stress. As can be seen, the maximum contact stress predicted by FEM (i.e., 2914 MPa) is in a very good agreement with the result of the Hertzian theory (i.e., 2890 MPa). The relative error for the maximum contact stress calculated by FEM and Hertzian theory is 0.83%.

## Results and discussion

### Defect parallel to the ring surface ($\user2{\varphi } = 0^\circ$)

The rolling of the ball on the ring surface was simulated by rotating the inner ring from $\theta = - \;8^\circ$ to $\theta = + \;8^\circ$ with two-degree steps. Out of this range, $\left| \theta \right| > 8^\circ$, the defect is located out of the subsurface contact stress filled. The stress intensity factors were determined by considering four contours for each crack (see Fig. 3b). Except for the first contour, whose results are usually incorrect, the values of stress intensity factors in other contours should be close. This closeness, in turn, can indicate the validity of the finite element model of the crack. Table 2 presents mixed mode stress intensity factors calculated by contours 2, 3, and 4 for cracks 1 and 3 for θ = 0°. These cracks have the same stress intensity factors as cracks 2 and 4, respectively. As can be seen, the difference in the values obtained from different contours is very small. Therefore, in addition to validating the finite element model of the cracks, the values obtained from any contour can be used. In the present study, the results of contour 3 are used to present the stress intensity factors of the cracks.

**Table 2:** Stress intensity factors of cracks 1 and 3, calculated by contours 2, 3, and 4 ($\theta =0^\circ$).

		Contour 2	Contour 3	Contour 4
Crack 1	${\text{K}}_{{\text{I}}}$	− 6.53	− 6.67	− 6.83
	${\text{K}}_{{{\text{II}}}}$	2.10	2.04	1.97
	${\text{K}}_{{{\text{III}}}}$	0.161	0.165	0.167
Crack 3	${\text{K}}_{{\text{I}}}$	− 1.84	− 1.91	− 1.98
	${\text{K}}_{{{\text{II}}}}$	0.0534	0.0565	0.0605
	${\text{K}}_{{{\text{III}}}}$	− 0.077	− 0.088	− 0.096

**Figure 8:**
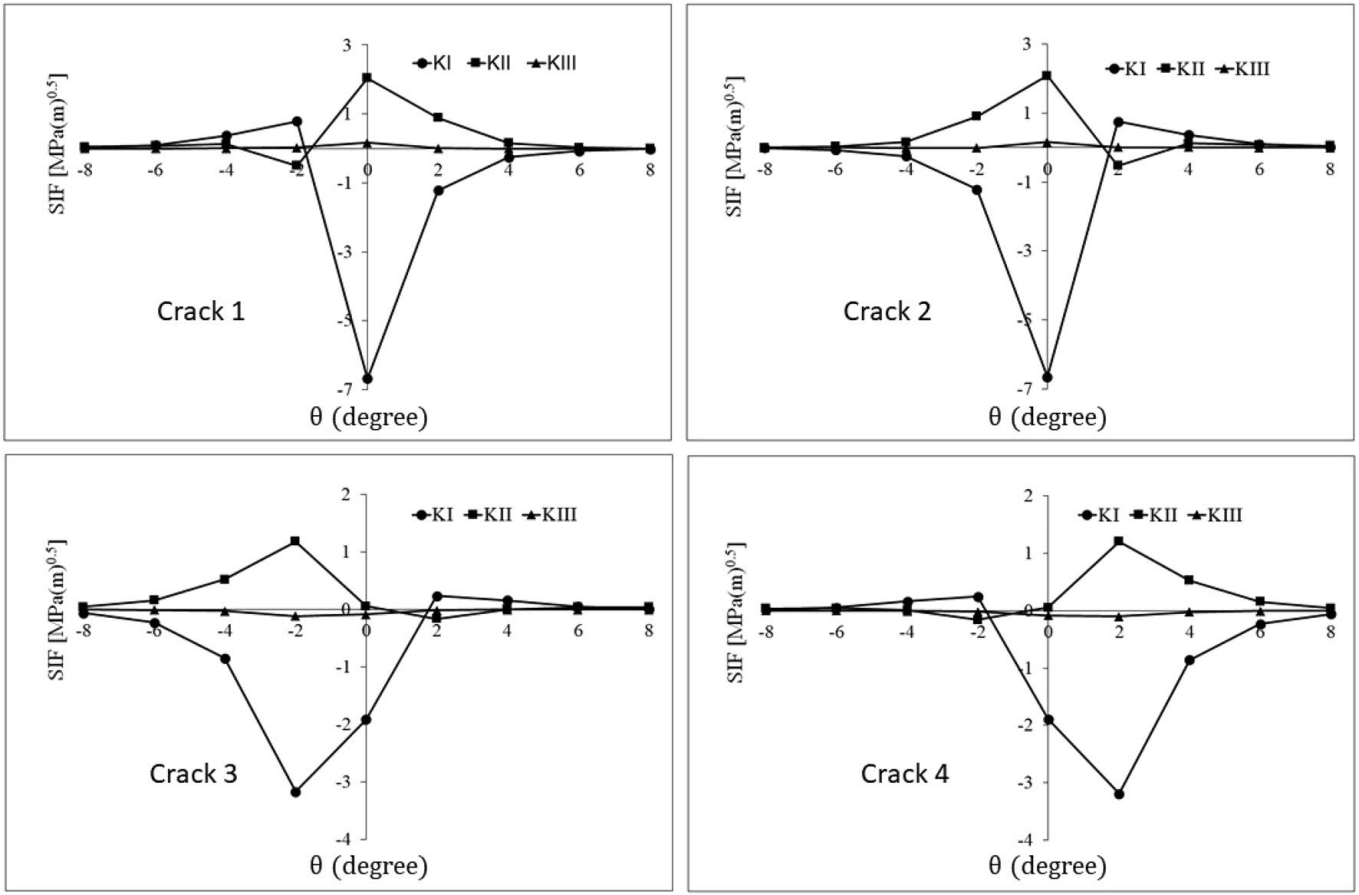
The history of mixed mode stress intensity factors for quadruplet cracks of the parallel defect to the contact surface of the ring.

Figure 8 exhibits the mixed mode stress intensity factors of the parallel pore to the surface of the ring groove. Looking at this figure, one can observe that the SIFs graph of crack 2 is the mirror of crack 1 diagrams with respect to the $\theta =0^\circ$ axis. A similar condition is available for the graphs of cracks 3 and 4. According to the geometrical and loading symmetry of the problem, another proof for the validity of the obtained values is provided.

When the pore is exactly under the ball, ${\mathrm{K}}_{\mathrm{I}}$ becomes negative for all cracks (Fig. 8, $\theta =0^\circ$); this is ascribed to very high compressive stress field of the ball/ring contact. This is an indication of the fact that under these conditions, the subsurface cracks or defects are not subjected to tensile mode loading. Instead, the faces of the cubic defect, (i.e., the two surfaces of each crack) come closer together rather than opening up. For shearing modes, modes II and III, the negative/positive sign of the calculated stress intensity factor only indicates the shearing direction. Hence, the effect of negative values of the shearing stress intensity factor is as destructive as their positive values. As the ball starts clockwise rolling from $\theta =- 8^\circ$, the cubic pore deforms to a parallelepiped due to the contact pressure. This deformation, which is consistent with the results of Ref.^9^, causes the opening of cracks 1 and 4 and consequently, tensile mode crack growth (positive ${\mathrm{K}}_{\mathrm{I}}$ in Fig. 8). Simultaneously, the closing of cracks 2 and 3 takes place, leading to the negative sign of ${\mathrm{K}}_{\mathrm{I}}$ in Fig. 8. By passing $\theta =0^\circ$, when the ball rolls toward the $\theta =+ 8^\circ$; this condition is completely reversed for all cracks.

For mode II SIFs graph, a peak appears in $\theta =0^\circ$ for cracks 1 and 2. This observation can be attributed to the maximum compressive stresses applied on the upper face of the pore in $\theta =0^\circ$, causing the crack front to undergo sliding displacement while tending to close. Also, mode II SIF reaches its maximum value for crack 3 at $\theta =- 2^\circ$ and for crack 4 at $\theta =+ 2^\circ$, which can be ascribed to the maximum subsurface shearing stress. The maximum values of mod II SIF for top cracks are greater than those for down cracks. Also, for the symmetry planes of the ball, pore, and ring to coincide, the tearing mode of SIF for all cracks is very small and insignificant (small ${\mathrm{K}}_{\mathrm{III}}$ values in Fig. 8). Finally, in contrast to classical fatigue and most of the cracks^25^, mode I is not the dominant mode of crack growth for rolling contact fatigue of the material defect, and sliding mode is the main fracture mode. This is conformity with the results reported by Brown and Miller’s work^26^. Moreover, closer cracks to the surface (cracks 1 and 2) have more suitable conditions for growth, and crack growth towards the rolling surface is more likely.

### The effect of defect orientation

**Figure 9:**
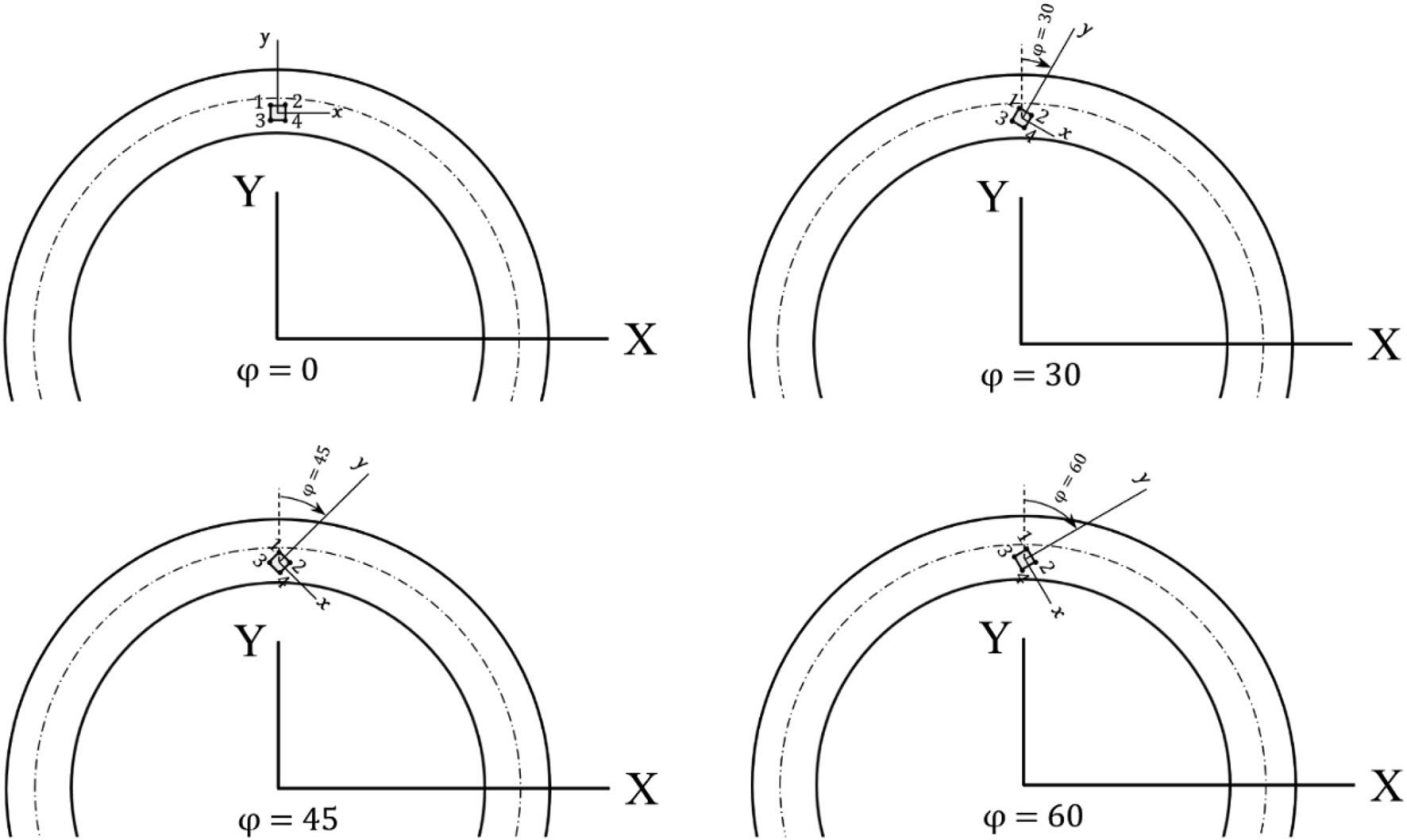
Defect orientations with respect to the contact surface, *φ* = 0°, 30°, 45°, and 60°.

In accordance with the findings from the parallel defect analysis, it is likely that crack initiation and propagation take place from the top face of the material defect. Nevertheless, the influence of defect orientation on the initiation and propagation of cracks remains unclear. Consequently, there is a need to examine the impact of pore orientation on Stress Intensity Factors (SIFs) to gain a better insight of its effect on crack behavior and initiation. Therefore, studying the pore orientation effect on SIFs is desirable. In this section, the problem is solved for the non-parallel defect considering the rotation angles *φ* = 30°, 45°, and 60° for the defect (see Fig. 9). In non-parallel defects, the size of the parallel defect and the depth of the parallel defect center from the groove surface is kept constant.

**Figure 10:**
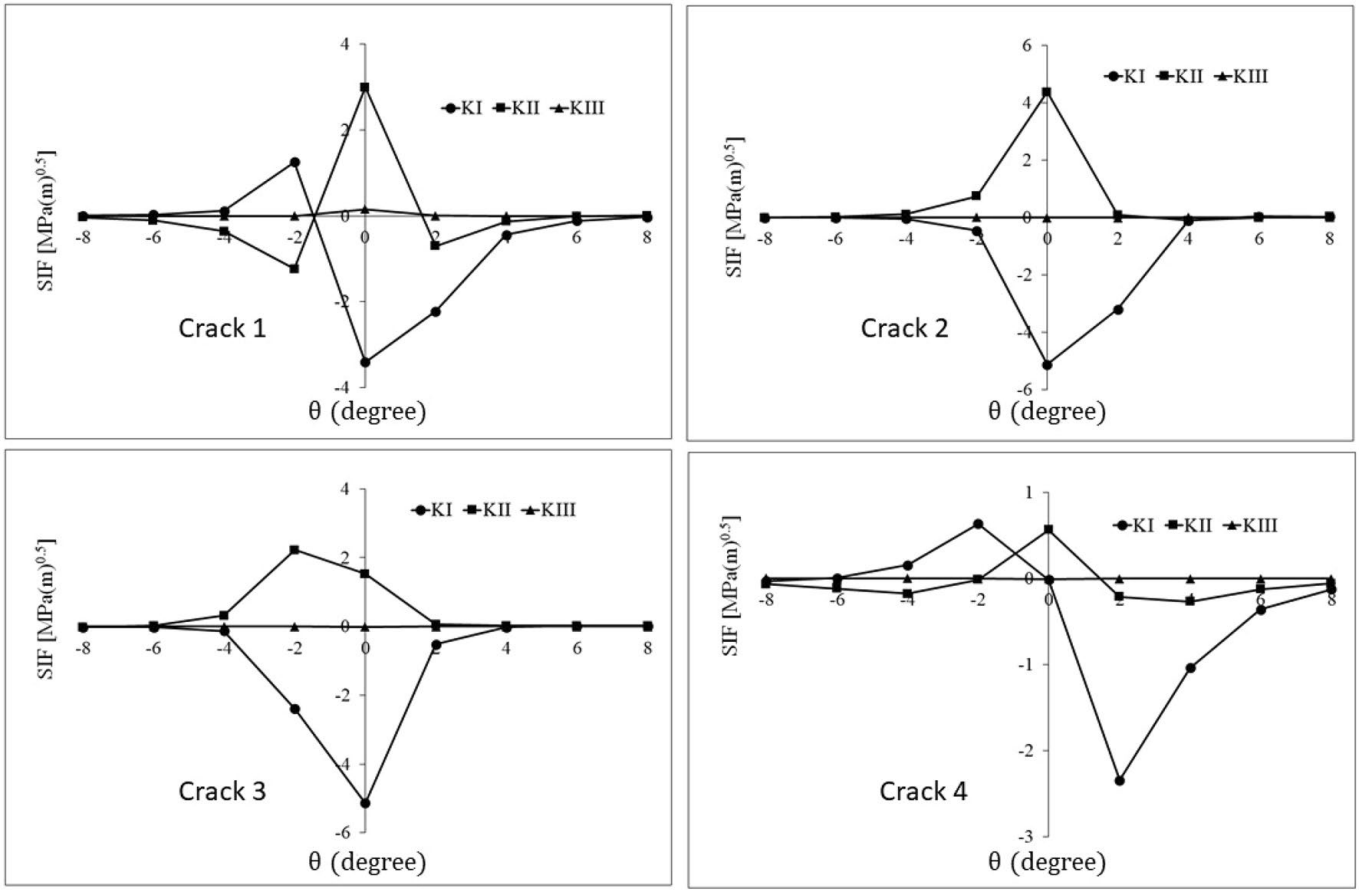
History of stress intensity factors of cracks 1–4 for *φ* = 30°.

**Figure 11:**
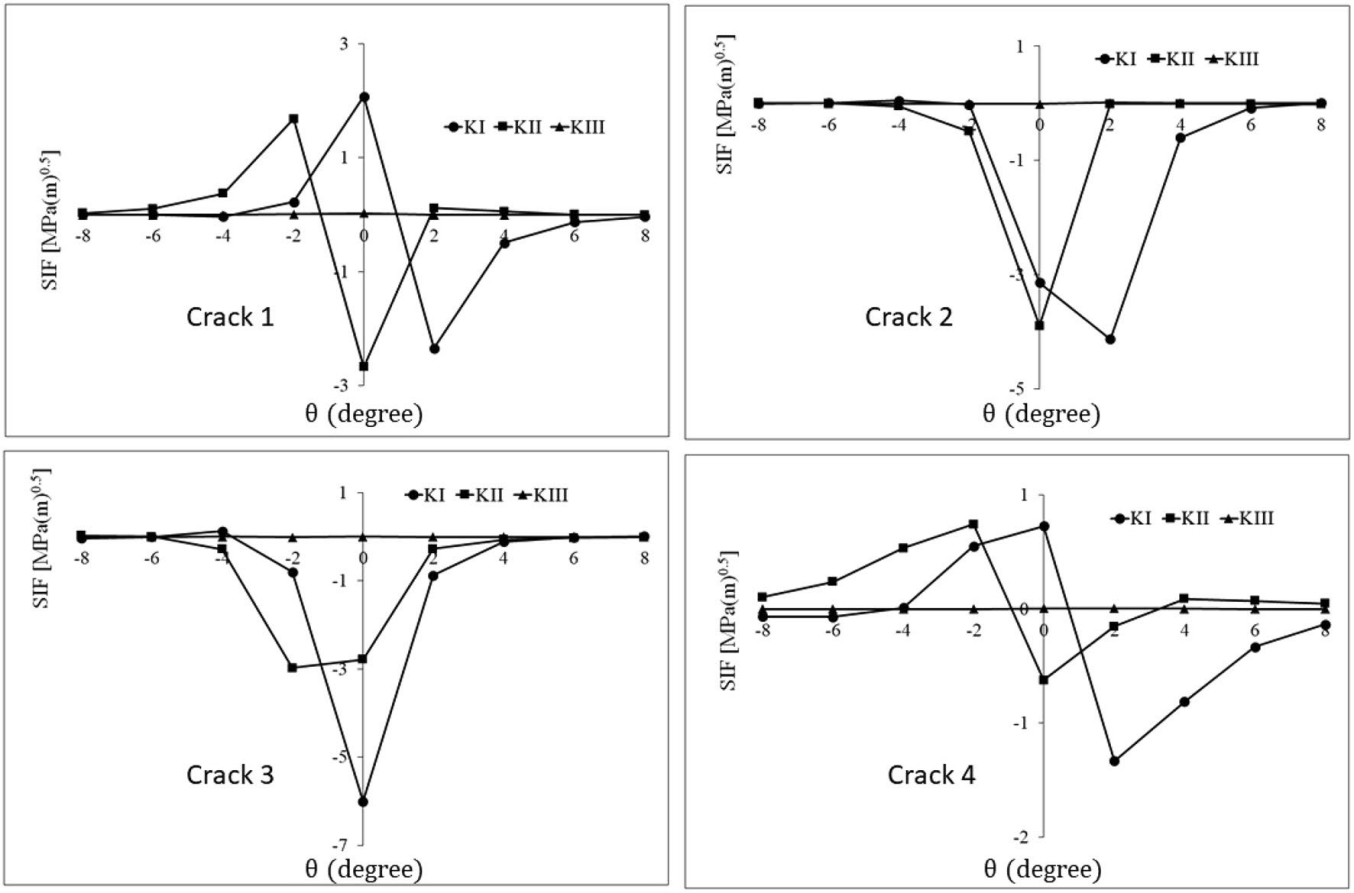
History of stress intensity factors of cracks 1–4 for *φ* = 45°.

**Figure 12:**
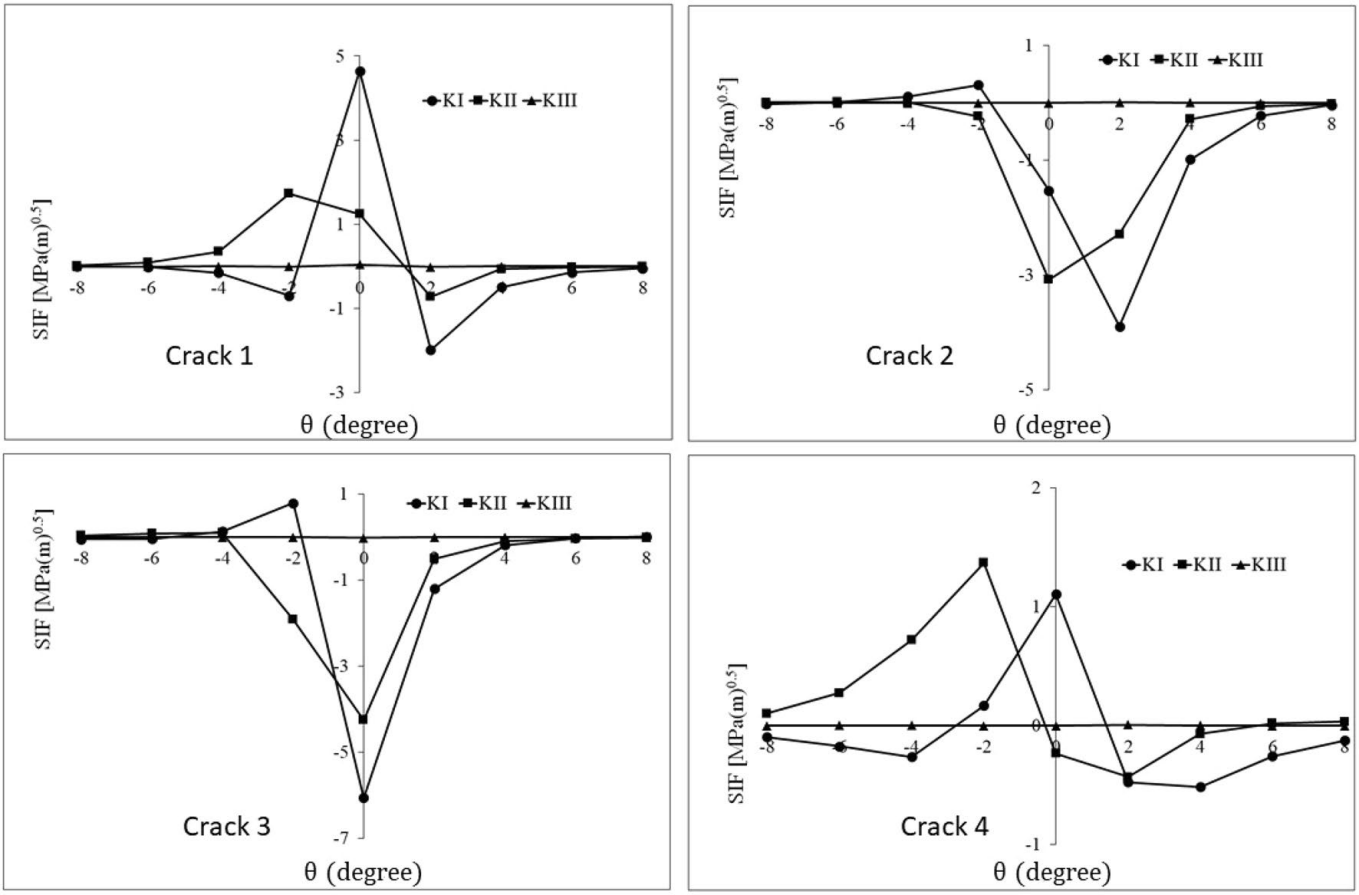
History of stress intensity factors of cracks 1–4 for *φ* = 60°.

Mixed mode stress intensity factors of the cracks for the mentioned defect orientations are calculated using FE analysis (Figs. 10, 11, 12). Comparing the Stress Intensity Factors (SIFs) of cracks for different defect orientations and analyzing the underlying causes for the observed variations can be a complex and challenging task. This challenge is due to the interaction of multiple parameters including rolling contact, defect and its cracks, direction of the crack surfaces, and the position of the crack tip concerning the contact stress field. Furthermore, the range of stress intensity factors plays the main role in fatigue crack growth. Thus, to evaluate the orientation effect of the material defect on the subsurface crack initiation and propagation potential, the values of the mixed mode stress intensity factor range for different orientations are calculated (Table 3). The range of stress intensity factors is calculated using FE analysis results of Figs. 10, 11 and 12 and Eq. (6):

6 $$\Delta K = K_{{{\text{max}}}} - K_{{{\text{min}}}}$$

**Table 3:** The range of stress intensity factors of cracks 1–4 for different orientations of the material defect.

Crack number	${{\varphi }}$	$\Delta {\text{K}}_{{\text{I}}} \left( {{\text{MPa}}\sqrt {\text{m}} } \right)$	$\Delta {\text{K}}_{{{\text{II}}}} \left( {{\text{MPa}}\sqrt {\text{m}} } \right)$	$\Delta {\text{K}}_{{{\text{III}}}} \left( {{\text{MPa}}\sqrt {\text{m}} } \right)$
Crack 1	0	0.79	2.54	0.169
	30	1.25	4.22	0.160
	45	2.07	4.35	0.031
	60	4.64	2.45	0.056
Crack 2	0	0.751	2.60	0.163
	30	0.043	4.48	0.010
	45	0.051	3.90	0.023
	60	0.312	3.09	0.012
Crack 3	0	0.232	1.34	0.114
	30	0.015	2.23	0.015
	45	0.126	3.00	0.015
	60	0.795	4.35	0.016
Crack 4	0	0.24	1.36	0.104
	30	0.63	0.83	0.005
	45	0.72	1.36	0.008
	60	1.10	1.80	0.010

As mode I SIF does not contribute to the growth of the closed crack, negative mode I SIF is considered zero (i.e., $K_{{{\text{min}}}} = 0$).

As can be seen in Table 3, the values of $\Delta {K}_{III}$ for all cracks and all orientations are very small and negligible. Also, by increasing the $\varphi$ from 0° to 60°, $\Delta {K}_{I}$ increases significantly for crack 1. However, for this crack, the changes in the range of mode II stress intensity factor are not monotonic as they first increase and then decrease. Therefore, in the case of crack 1, it can be stated that the orientation of the defect has a significant effect on its growth condition, and the dominant crack growth modes are I and II.

For crack 2, mode I SIF ranges are negligible compared to mode II SIF ranges. Since the value of shearing stress on the crack surfaces changes by changing the defect orientation, the range of mode II stress intensity factor is affected by the defect orientation and is maximum for *φ* = 30°. Furthermore, the insignificant values of $\Delta {K}_{I}$ decrease with the clockwise rotation of the defect. Under contact pressure, due to the rectangular parallelepiped geometry of the defect, an increase in $\varphi$ leads to an increase in the opening mode of cracks 1 and 4 and the closing mode of cracks 2 and 3. In addition, irregular changes in the mode I stress intensity factor range (for cracks 2 and 3) can be considered as the interaction of two effects. The first one is the effect of material defect orientation changing on the displacement field of crack surfaces. The second is the effect of the material defect orientation changing on the getting further away/closer of the crack front from/to the subsurface contact stress field. Finally, in the case of crack 2, it can be stated that the change of martial defect orientation affects the state of the defect, and the dominant mode for crack growth is mode II.

For crack 3, similar to crack 2, mode I is very insignificant compared to mode II. Also, the range of mode II stress intensity factor always increases with the defect orientation change; the increase is more than threefold. Additionally, the negligible values of $\Delta {K}_{I}$ change irregularly with the rotation of the defect because of the same reason mentioned for mode I of crack 2. Therefore, in crack 3, the defect orientation imposes a significant effect on its conditions, and the dominant mode of crack growth is mode II.

In the case of crack 4, the values of $\Delta {K}_{I}$ mainly increase with the increase of $\varphi$. In general, the stress intensity factor range of crack 4 has lower values than other cracks. This difference can be attributed to the greater distance of this crack from the subsurface stress field, especially in non-parallel orientations. Thus, crack 4 has less importance in comparison with the others.

### Tanaka model

In classical fatigue, the tensile fracture mode is a determinant for the crack experiencing mixed fracture modes. But in rolling contact fatigue, where the tensile fracture mode has less importance, the contribution of the shearing fracture modes is also important in determining the crack condition. In section “The effect of defect orientation”, it was observed that the change of material defect orientation, while leading to an increase in the range of SIFs in one fracture mode, causes a decrease in the range of SIFs in the other fracture mode. Therefore, this section aims to assess the crack condition under the increasing and decreasing behavior of SIFs range in different modes by determining the equivalent range of SIFs for different crack orientations. In other words, the definition of the equivalent SIF is usually used to evaluate a crack condition under mixed fracture modes. In this section, using the Tanaka model^27^ for different orientations of the cracks, the equivalent SIF range is calculated and given in Table 4. Tanaka model only needs Poisson’s ratio of the material to calculate the range of the equivalent stress intensity factor (${\Delta K}_{{{\text{eq}}}}$),

7 $${\Delta }K_{eq} = \left[ {\Delta K_{I}^{4} + 8\Delta K_{II}^{4} + \frac{8}{1 - \nu }\Delta K_{III}^{4} } \right]^{0.25}$$

**Table 4:** The equivalent range of stress intensity factor for cracks 1–4.

Crack number	${{\varphi }}$	$\Delta {\text{K}}_{{{\text{eq}}}} \left( {{\text{MPa}}\sqrt {\text{m}} } \right)$
Crack 1	0	4.28
	30	7.11
	45	7.33
	60	5.24
Crack 2	0	4.38
	30	7.54
	45	6.56
	60	5.20
Crack 3	0	2.26
	30	3.76
	45	5.05
	60	7.32
Crack 4	0	2.29
	30	1.41
	45	2.30
	60	3.04

As shown in Table 4, the maximum value of the equivalent stress intensity factor range for cracks 1, 2, and 3 are close to each other, while the maximum equivalent stress intensity factor range for crack 4 is less than half of the other cracks. The maximum value of equivalent stress intensity factor range among all cracks and directions is related to crack 2 with $\varphi = 30^\circ$ and $\Delta K_{eq} = 7.54 \;{\text{MPa}}\sqrt {\text{m}}$. In this orientation, cracks 1 and 2 have a high and almost the same propagation probability. Also, cracks 3 and 4 at $\varphi = 60^\circ$ have the maximum equivalent range of stress intensity factor and the highest crack growth probability. In the case of $\varphi = 0^\circ$, the equivalent stress intensity factor range of the top cracks near the contact surface is almost twice that of the down cracks. Also, the crack most affected by the defect orientation is crack 3, whose range of equivalent stress intensity factor at $\varphi = 60^\circ$ is 3.2 times larger than that in the state of the parallel defect. This difference highlights that the effect of the orientation of the material defect on the potential of crack initiation and propagation from its location is significant and must be considered in the relevant studies.

## Conclusions

This study developed and validated a three-dimensional finite element model of a bearing ring containing a material defect below the raceway surface. To this end, the stress intensity factors of the material defect were modeled and investigated in the form of a cube with four cracks. The history of the cracks' mixed mode stress intensity factor was calculated for different orientations of the material defect. Then, the range of mode I, II, and III stress intensity factors and Tanaka equivalent stress intensity factor range were determined, compared, and evaluated. Based on the results, the following conclusions can be drawn:

1. The orientation of the material defect has an important effect on the defect capability in crack initiation and growth. This effect in the studied range can increase the opening mode of the SIF range, the most effective mode in crack growth, more than 5 times (crack 1 at the orientation of $\varphi = 60^\circ$ compared to $\varphi = 0^\circ$). Moreover, the change of the defect orientation can increase more than 3 times the equivalent stress intensity factor ranges.
2. The defect tendency for crack initiation and growth from its location is changed by altering the direction of the defect by changing the defect planes with respect to the subsurface contact stress field, and also changing the distance of the crack fronts from the contact point and as a result, the intensity of the stress field.
3. For the subsurface defect, ${\text{K}}_{{{\text{III}}}}$ values are insignificant compared to the mode I and II SIFs and have a negligible effect on crack growth. Certainly, this point can be different if the defect is not exactly under the contact area or its orientation is such that the crack fronts are not parallel to the bearing axis.
4. The dominant crack growth mode in the subsurface defect is the shearing mode II. This point, along with the maximum subsurface shearing stresses in rolling contact, makes subsurface material defects prone to damage growth from their location and the final fracture of rolling contact structures.
5. In a considerable part of the SIF history, the ball rolling on the bearing ring, cracks are compressed, and mode I does not affect their growth.

## Data Availability

All data generated or analyzed during this study are included in this published article.

## List of symbols

$a$: Semi-major axis of contact ellipse (mm)
$b$: Semi-minor axis of contact ellipse (mm)
$H$: Depth of the material defect (mm)
$K_{I}$: Mode I stress intensity factor $\left( {{\text{MPa}}\sqrt {\text{m}} } \right)$
$K_{II}$: Mode II stress intensity factor $\left( {{\text{MPa}}\sqrt {\text{m}} } \right)$
$K_{III}$: Mode III stress intensity factor $\left( {{\text{MPa}}\sqrt {\text{m}} } \right)$
$\Delta K$: Stress intensity factor range $\left( {{\text{MPa}}\sqrt {\text{m}} } \right)$
$\Delta K_{eq}$: Equivalent range of stress intensity factor $\left( {{\text{MPa}}\sqrt {\text{m}} } \right)$
$R_{1} \;\& \;R^{\prime}_{1}$: The radii of the main curves of the ball (mm)
$R_{2} \& R^{\prime}_{2}$: The radii of the main curves of the ring (mm)
$\varphi$: Material defect orientation ($^\circ$)
$\sigma_{max}$: Maximum contact stress (${\text{MPa}}$)
$\theta$: Rotation angle of the ball on the bearing ring ($^\circ$)
